# Atomic force and infrared spectroscopic studies on the role of surface charge for the anti-biofouling properties of polydopamine films

**DOI:** 10.1007/s00216-022-04431-7

**Published:** 2022-11-24

**Authors:** Giada Caniglia, Andrea Teuber, Holger Barth, Boris Mizaikoff, Christine Kranz

**Affiliations:** 1 Institute of Analytical and Bioanalytical Chemistry, Ulm University, Albert Einstein Allee, 11, 89081 Ulm, Germany; 2 Institute of Pharmacology and Toxicology, University of Ulm Medical Center, Albert Einstein Allee, 11, 89081 Ulm, Germany; 3 Hahn-Schickard, Sedanstraße 14, 89077 Ulm, Germany

**Keywords:** Biofilm, Polydopamine, Electrodeposition, Point of zero charge, Atomic force microscopy (AFM) force spectroscopy, Attenuated total reflection-Fourier transform infrared (ATR-FTIR) spectroscopy

## Abstract

Antibacterial polymer materials have gained interest due to their capability to inhibit or eradicate biofilms with greater efficiency in comparison with their monomeric counterparts. Among the antimicrobial and anti-biofouling polymers, catecholamine-based polymers — and in particular polydopamine — have been studied due to their favorable adhesion properties, which can be tuned by controlling the pH value. In this study, we used atomic force microscopy (AFM)–based spectroscopy to investigate the relation between the adhesion properties and surface charge density and the pH of electrochemically deposited polydopamine films presenting a dissociation constant of polydopamine of 6.3 ± 0.2 and a point of zero charge of 5.37 ± 0.06. Furthermore, using AFM and attenuated total reflection-Fourier transform infrared spectroscopy (ATR-FTIR), the influence of the surface charge density of polydopamine on bacterial adhesion and biofilm formation was investigated. It was shown that the adhesion of *Escherichia coli* at positively charged polydopamine is three times higher compared to a negatively charged polymer, and that the formation of biofilms is favored at positively charged polymers.

**Graphical abstract:**

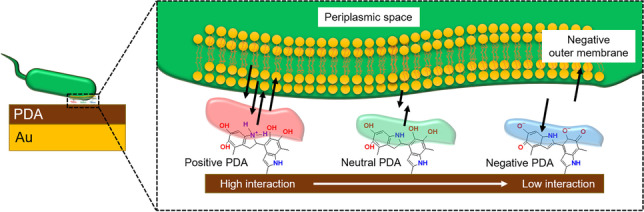

**Supplementary Information:**

The online version contains supplementary material available at 10.1007/s00216-022-04431-7.

## Introduction

In the last decades, antimicrobial and anti-biofouling polymers (AMPs) [1] have gained considerable interest due to their low toxicity, biocompatibility, and efficiency to eradicate or inhibit biofilms and the proliferation of antibiotic-resistant bacteria. The antimicrobial efficiency of AMPs arises from the presence of biocidal functional groups such as quaternary nitrogen groups, tertiary amines, halamines, and catechol groups [2, 3]. Polydopamine (PDA) — a mussel-inspired catecholamine-based polymer — is classified as an AMP, and its anti-biofouling property is exploited, e.g., for filtration membranes [4]. PDA is known to exhibit distinct mechanical, physicochemical, and electrical properties that can be tuned by the deposition method and the experimental conditions [5–7]. PDA films are mainly obtained by dip-coating via oxidation and self-polymerization in basic dopamine solutions (pH > 7.4). This method was first described by Messersmith and coworkers [8] and has been widely studied [9], as any surface can be coated by PDA via dip-coating. However, this strategy is of limited control on the oxidation state, homogeneity of the deposited films at short deposition times, conductivity, and thickness of the obtained polymer film [10]. In contrast, electrochemical methods such as cyclic voltammetry [11, 12] and pulsed deposition techniques [10, 13–15] have shown substantial potential to readily control and tune the physicochemical properties of PDA. Even though there is a significant body of literature focused on the chemical and mechanical properties of electrochemically deposited PDA (e-PDA) [11, 16–19], the anti-biofouling properties and the interaction of bacteria with e-PDA films are still a little explored field [15, 18, 20, 21]. It has been shown that bacterial adhesion is strongly influenced by the oxidation state of e-PDA with increased bacterial adhesion, if the polymer is in its oxidized form [15]. However, bacterial adhesion is also controlled by the surface charge of the polymer, which can be modified by varying the pH and the ionic strength of the medium the PDA-coated sample is immersed. The relation of the surface charge density with the pH and the point of zero charge (PZC) of PDA films obtained by dip-coating strongly depends on the deposition conditions of the polymer, as the PZC varies from PZC = 4.00 if PDA is synthesized in the presence of Tris-buffer [22] to PZC = 6.50 when formed with copper(II) as an oxidant [23]. To date, studies related to the surface charge density of e-PDA and its interactions with bacterial cells remain limited.

In the present work, we investigate the first stages of attachment of bacteria at pulse-deposited e-PDA films in dependence on the pH via AFM-based force spectroscopy [24, 25] and attenuated total reflection-Fourier transform infrared (ATR-FTIR) spectroscopy [26–32]. Also, studies via AFM force titration on the adhesive properties of e-PDA as a function of the pH were performed [33–36] to determine the dissociation constants of the polymer.

## Materials and methods

### Reagents and materials

All solutions were freshly prepared with deionized water (18.0 MΩ cm, Elga Labwater; VWR Deutschland, Germany). Dopamine hydrochloride was purchased from Sigma-Aldrich (Germany). Luria–Bertani (LB) culture medium was purchased from VWR International GmbH (Germany). Sodium chloride (NaCl), potassium chloride (KCl), sodium hydrogen phosphate (Na_2_HPO_4_), sodium dihydrogen phosphate (NaH_2_PO_4_), hydrochloric acid (HCl), sodium hydroxide (NaOH), potassium hydroxide (KOH), and ferrocene-methanol were purchased from Merck (Germany). 2 cm × 2 cm gold substrates were prepared by sputter coating on silicon wafers and were cleaned in acetone, isopropanol, and deionized water, prior to use. All electrochemical experiments were performed using a CHI842B bipotentiostat (CH Instruments, USA) in a three-electrode cell with an Ag/AgCl/KCl (sat.) reference electrode, a platinum counter electrode, and the gold substrate as a working electrode.

### Electrodeposition of polydopamine

The deposition of e-PDA was done as previously described [10, 15]. Briefly, e-PDA films were deposited onto gold-coated substrates applying 100 pulse cycles with a potential pulse sequence of + 0.5 V/2 s; 0.0 V/2 s; − 0.3 V/2 s; 0.0 V/3 s vs. Ag/AgCl/KCl (sat.). The deposition was performed in freshly prepared and with argon purged 5.3 mmol L^−1^ dopamine hydrochloride solution in 10 mmol L^−1^ PBS (pH 7.4). Once e-PDA was pulsed-deposited onto the gold-coated substrate, the polymer was further electro-oxidized in a solution of 10 mmol L^−1^ PBS by applying a potential of + 0.5 V during 300 s vs. Ag/AgCl/KCl (sat.).

### Bacterial culture conditions

*Escherichia coli* strain DH5-α (originally obtained from Clontech Laboratories, Inc., Heidelberg, Germany) cultures were prepared by inoculating 25 g L^−1^ sterile LB medium at 37 ± 1 °C up to a concentration of 10^9^ CFU mL^−1^. Bacterial growth was monitored by the OD_600_ using a UV–VIS spectrometer (Thermo Scientific NanoDrop One, MA, USA). The bacterial suspension was then harvested and resuspended in dilute LB medium (0.5 g L^−1^) with different pH, in a range of pH 5 to 7. The pH of the LB medium was adjusted by adding dilute HCl or NaOH and monitored using a pH meter (827 pH Lab, Metrohm, Switzerland). The culture was seeded on the e-PDA samples by immersing them into the bacterial solution and incubating at 37 ± 1 °C, using a shaking incubator at a speed of 1.0 s^−1^ (KS 4000ic control, Keison Products, UK) for 16 h. For the bacteria adhesion experiments, the e-PDA samples were rinsed twice with LB medium and twice with MilliQ water and immersed in a 10 mmol L^−1^ PBS (pH 7.4) solution.

### Force-distance measurements and AFM imaging

AFM measurements were performed using a 5500 AFM/SPM microscope (Keysight Technologies, AZ, USA) equipped with a closed-loop scanner. AFM contact mode images were recorded in air using silicon nitride probes (MLCT, Bruker AFM probes, CA, USA; nominal spring constant of 0.1 N m^−1^) and a scan speed of 0.64 ln s^−1^. Force titration experiments and bacterial adhesion experiments were performed via AFM-based force spectroscopy. Force-distance curves were recorded in solution using silicon nitride probes (MLCT, Bruker AFM probes, CA, USA; tip radius of 20 nm and nominal spring constant of 0.1 or 0.6 N m^−1^) with a sweep rate of 1.0 µm s^−1^ to minimize hydrodynamic effects and a loading force of 200 nN. The force constants of the cantilevers were determined using the thermal noise method [37]. Force titrations on e-PDA were carried out in MilliQ water with the pH adjusted by adding HCl or NaOH, while bacterial adhesion measurements were performed in 10 mmol L^−1^ PBS (pH 7.4). For the charge density measurements, the AFM probes were treated with UV-ozone, prior to each experiment. Statistical analyses are based on the Student *t*-test assuming unequal variance. MoutainSPIP® v. 9 (Digital Surf, France) and OriginPro® 2019b software, V 9.6.5.169 (Origin Lab Corporation) were used to obtain the values of adhesion forces and surface charge.

### ATR-FTIR spectroscopy

The ATR-FTIR experiments were done using a Fourier transform infrared (FTIR) spectrometer (Alpha II, Bruker Optics GmbH, Germany) equipped with an ATR assembly (Platinum ATR, Bruker Optics GmbH, Germany) providing a single-bounce diamond crystal serving as an internal reflecting element (IRE). Six different e-PDA samples were investigated via ATR-FTIR, whereby for each pH value, two e-PDA samples were prepared: (i) an e-PDA sample inoculated with *E. coli*, and (ii) an e-PDA sample only exposed to the LB medium. Each sample was rinsed twice with LB medium and twice with MilliQ water. For the e-PDA samples inoculated with *E*. *coli*, the corresponding e-PDA samples only immersed in LB medium were recorded as the background spectrum. IR spectra were recorded at a spectral resolution of 2 cm^−1^ averaging 64 scans using the OPUS software package (Bruker Optics GmbH, Germany). Data treatment was conducted using a 7-point FFT filter (Origin 2019b, OriginLab). No baseline correction was applied.

## Results and discussion

### Characterization of e-PDA

#### Pulse deposition and force titration of e-PDA

e-PDA films were pulse-deposited on a gold substrate and after deposition, a positive potential (+ 0.5 V vs. Ag/AgCl) was applied for 300 s to oxidize phenolic moieties of the PDA film. Figure S1a shows the current response of the e-PDA-modified electrode during the first and last cycles of the pulsed deposition. A decrease in the current (Fig. S1a) is due to the formation of the non-conductive polymeric film which reduces the accessibility of the electroactive species (ferrocene-methanol) to the gold electrode, blocking the charge transfer. The insulating nature of the PDA film is also confirmed by the cyclic voltammogram recorded before (Fig. S1b, black line) and after (Fig. S1b, red line) the modification of the gold electrode.

According to the literature [17, 38], the mechanism of polymerization of dopamine follows an electrochemical-chemical-electrochemical (ECE) pathway. The proposed mechanism (Fig. S2) consists of the first electro-oxidation of dopamine, in which a two-electron two-proton reaction takes place, converting the *o*-diphenolic group of dopamine to dopamine-quinone (DAQ, $E^{\circ\prime}=$ + 0.2 V vs. Ag/AgCl/KCl, sat.) [39]. The second step involves intramolecular cyclization via Michael addition of DAQ with the formation of leucodopaminochrome (LDAC). The final electrochemical step leads to the formation of dopaminechrome (DAC, $E^\circ =$ −0.3 V vs. Ag/AgCl/KCl, sat.) [39]. Once DAC is formed, other isomerization processes occur leading to, e.g., the formation of indole motifs. After the formation of e-PDA herein, an additional oxidation step at + 0.5 V was applied to ensure the oxidation of the *o*-diphenols to obtain *o*-quinone motifs ($E^{\circ\prime}=$ + 0.2 V vs. Ag/AgCl/KCl, sat.) [39]. Most mechanistic studies of polydopamine have been reported for PDA obtained via dip-coating [9, 40]. In recent years, secondary pathways during the electrochemical polymerization of dopamine have been suggested, like the presence of a small amount of pyrrol-carboxyl groups based on X-ray photoelectron spectroscopy studies [41, 42], which might be formed via the oxidative degradation of indole-based species and the appearance of open-chain amines formed after the first oxidation step [41]. The latter suggests a pathway in which intramolecular cyclization does not take place. The proposed mechanisms of the electrochemical polymerization of PDA and the suggested products are illustrated in Fig. S2.

**Fig. 1:**
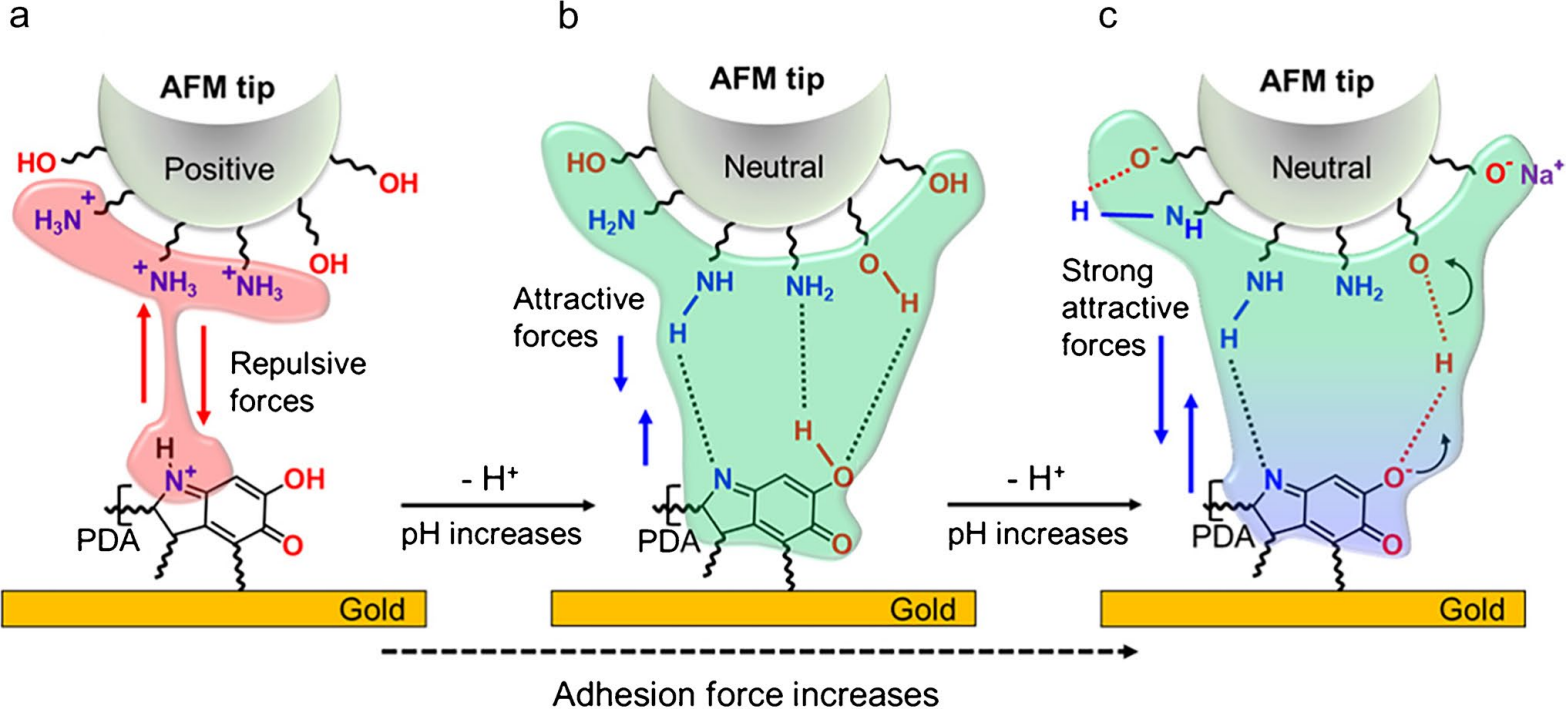
Schematics of the interactions between the AFM probe and the e-PDA substrate at (**a**) acidic pH (pH < 6), (**b**) neutral and slightly acidic pH (pH 6–7), and (**c**) slightly alkaline pH (pH > 8)

PDA is known for its excellent adhesion properties, which depend also on the experimental conditions [10, 43–45]. For instance, using AFM force spectroscopy, a strong dependence of the adhesion forces of pulse-deposited e-PDA has been observed at two different pH values, demonstrating a pronounced decrease in adhesion in acidic conditions (pH 3) [10]. Herein, we performed a detailed study of the adhesion forces in dependence on the pH (starting at pH 3 up to pH 10) by using the force titration method [35]. With this method, the magnitude of the adhesion forces is associated with the ionization states of both surfaces (AFM probe and e-PDA), as depicted in Fig. 1. An abrupt variation in the adhesion value at a certain pH is correlated with the dissociation constant of the polymeric surface.

**Fig. 2:**
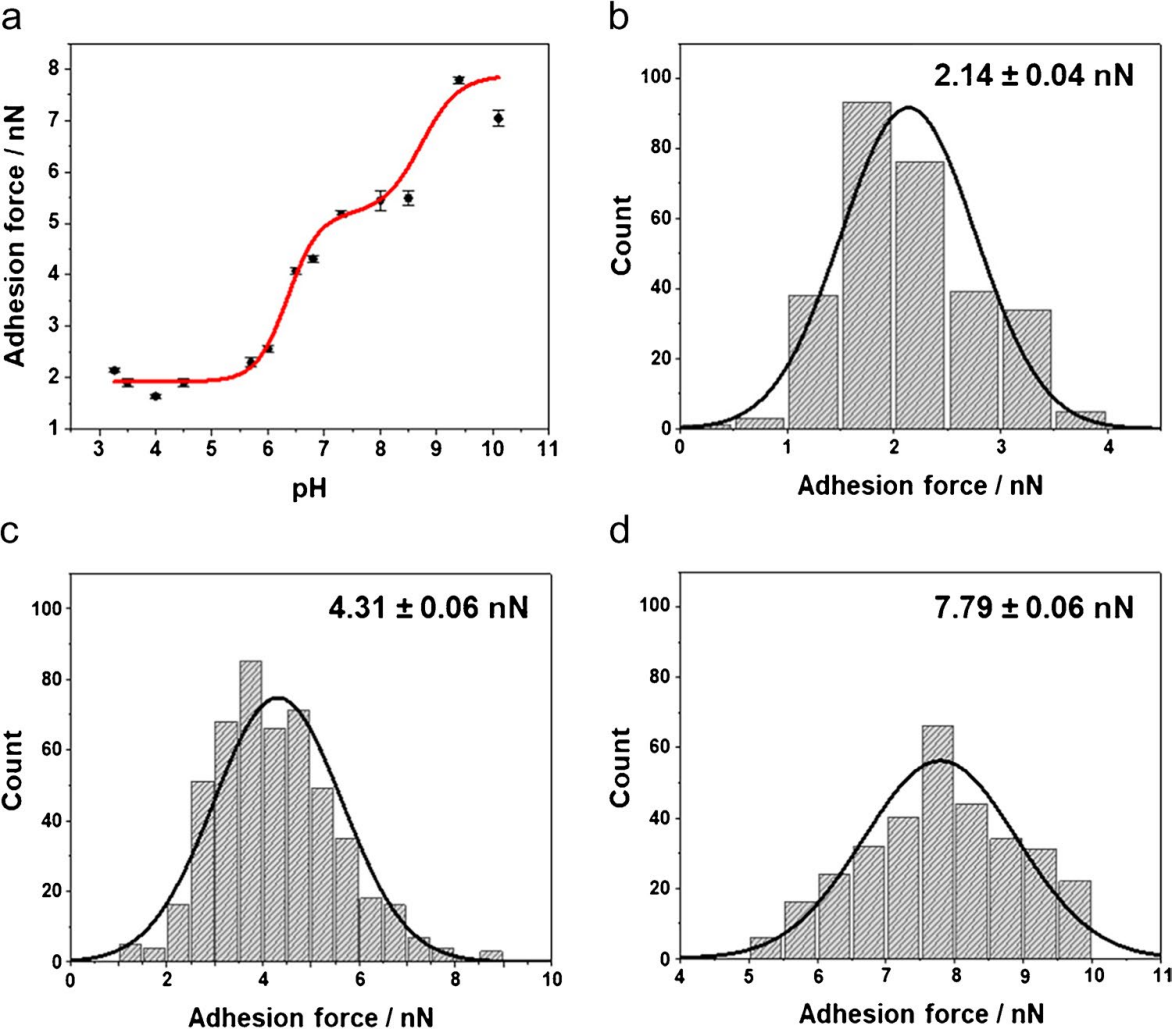
(**a**) Force titration of PDA immersed in different solutions with different pH values. Histograms of the adhesion forces at (**b**) pH 3.27, (**c**) pH 6.80, and (**d**) pH 9.40 (*n* = 300)

The force titration curve is shown in Fig. 2a, while Fig. 2b–d shows representative histograms of the adhesion forces at three different pH values. The high adhesion forces at alkaline pH (4.31 ± 0.06 nN, *n* = 500) and the sharp drop of the adhesion force at a pH close to pH = 9.0 and below pH = 7.0 can be explained by the attractive and repulsive forces between the AFM probe and the e-PDA surface (Fig. 1). These interactions arise from the protonation and deprotonation of the functional groups of both surfaces. For a better understanding of these interactions, the chemical nature of the AFM probe must be taken into account.

If the silicon nitride AFM probe (used in this study) is immersed in an aqueous solution, the tertiary amine Si_3_N produces silanol and amine sites according to the following reactions [46, 47]:

1 $${\mathrm{Si}}_{3}\mathrm{N}+{\mathrm{H}}_{2}\mathrm{O}\leftrightarrows {\mathrm{Si}}_{2}\mathrm{NH}+\mathrm{SiOH}$$

2 $${\mathrm{Si}}_{2}\mathrm{NH}+{\mathrm{H}}_{2}\mathrm{O}\leftrightarrows {\mathrm{SiNH}}_{2}+\mathrm{SiOH}$$

Since Reactions 1 and 2 are known to be kinetically very fast [46], it was assumed that the silanol and the secondary amine are the dominant species present at the AFM tip, and are responsible for the charge regulation of the AFM probe [46, 48], i.e., are involved in the adsorption and desorption of hydronium ions. Depending on the pH, silanol and amine groups may dissociate or protonate according to the following chemical equilibria [48, 49]:

3 $$\mathrm{SiOH}\leftrightarrows -{\mathrm{SiO}}^{-}+{\mathrm{H}}^{+}$$

4 $$\mathrm{SiOH}+{\mathrm{H}}^{+}\leftrightarrows {\mathrm{SiOH}}_{2}^{+}$$

5 $${\mathrm{SiNH}}_{2}+{\mathrm{H}}^{+}\leftrightarrows {\mathrm{SiNH}}_{3}^{+}$$

It has been shown by X-ray photoelectron spectroscopy that a significant amount of ${\mathrm{SiOH}}_{2}^{+}$ sites are present at a very low acidic pH (lower than 2), while the deprotonation of the silanol groups is expected at a slightly alkaline pH (higher than 8) [50]. Therefore, in the pH range investigated during the present studies (pH 3 to 10), the surface charge of the silicon nitride AFM probe may be influenced by the equilibria shown in Reactions 3 and 5, respectively (Fig. 1).

Thus, the low adhesion detected at acidic pH (Fig. 2a and b) is a consequence of a high electrostatic repulsion between the AFM probe and the e-PDA surface, due to the interaction between the $-{\mathrm{NH}}_{3}^{+}$ groups of the AFM probe and the positively charged groups derived principally from the protonation of indoline and indole motifs of the e-PDA (Fig. 1a), which $p{K}_{a}$ is approximately 5.5 [22, 51]. The significant increase in adhesion at pH values close to 6 is then due to the deprotonation of the amine groups of e-PDA and the silicon nitride AFM probe, decreasing the probe-sample repulsion, as shown in Fig. 1b. This change is also revealed by the higher dispersion of the adhesion values at a pH between 6 and 7 (broad histogram shown in Fig. 2c), which may be related to the presence of protonated and deprotonated groups. This abrupt change in adhesion is thus interpreted as an approximation to the $p{K}_{a}$ of the e-PDA surface groups, which is $p{K}_{a}$ = 6.3 ± 0.2 (first inflection point of the double Boltzmann sigmoidal fit in Fig. 2a). This data is consistent with the predicted dissociation constants of amine and imine groups present in the polymer ($p{K}_{a}=$ 5.8 for indoline-quinones and $p{K}_{a}=$ 5.4 for indole-quinones) [22, 51]. The slight variation of the dissociation constant obtained by the force titration ($p{K}_{a}$ = 6.3) in comparison to the predicted values ($p{K}_{a}=$ 5.8 and 5.4) is explained by the presence of deprotonated carboxyl and semiquinone groups of e-PDA ($p{K}_{a} =$ 3.6 and 4.7, respectively) [19]. These negatively charged functional groups contribute to the total probe-sample interaction, thereby increasing the adhesion force.

At pH values > 8 (Fig. 2a and d), a decrease in adhesion would have been expected due to the deprotonated silanol groups of the AFM probe, which would interact with the negatively charged quinone groups of the e-PDA surface. However, the interaction of the $-{\mathrm{SiO}}^{-}$ group with the counterions present in the solution (i.e., ${\mathrm{Na}}^{+}$ derived from the $\mathrm{NaOH}$ added to the solution) may reduce the concentration of the negatively charged silanol moieties [52]. The formation of hydrogen bonds between $-{\mathrm{SiO}}^{-}$, neutral $-\mathrm{SiOH}$, and $-{\mathrm{SiNH}}_{2}$ groups present on the surface of the AFM probe may further decrease the negative charges at the surface of the AFM probe and increase the electrostatic interaction with the e-PDA surface (Fig. 1c).

#### Point of zero charge of e-PDA

The surface charge density and the point of zero charge of e-PDA were obtained using AFM-based force spectroscopy [24, 25, 53]. If the AFM cantilever approaches a surface immersed in a solvent, the deflection response depends on the forces experienced by the tip. These forces consist of short- and long-range interactions including van der Waals, electrostatic (electrical double layer, EDL), and hydrodynamic interactions, and are a function of the probe-surface distance. For an AFM probe with a radius larger than 20 nm [54] (used in the presented studies) — i.e., larger than the Debye length of the system [53, 54] — the probe-surface system can be considered an ideal sphere-flat surface system. In this ideal system, the interaction forces are described using the Derjaguin, Landau, Verwey, and Overbeek (DLVO) theory [55], and the surface charge density of the sample and the AFM probe are extracted by solving the linearized Poisson-Boltzmann equation (PBE) [56]. In the present study, it is assumed that (i) the surface charge of the probe and the sample remains constant during the approach of the AFM probe (constant charge condition), and (ii) the major contribution arises from the EDL interaction, neglecting van der Waals forces since the probe-surface system was studied at a distance greater than the Debye length [57].

The surface charge density of the e-PDA was extrapolated by fitting the AFM force-distance approach curves with the PBE, shown in Eq. 1,

1 $${F}_{\mathrm{EDL}}=\frac{2\pi R{\lambda }_{D}}{\varepsilon {\varepsilon }_{0}}\left[2{\sigma }_{S}{\sigma }_{T}\mathrm{exp}\left(-\frac{d}{{\lambda }_{D}}\right)+\left({\sigma }_{S}^{2}+{\sigma }_{T}^{2}\right)\mathrm{exp}\left(-\frac{2d}{{\lambda }_{D}}\right)\right]$$

where $\varepsilon$ and ${\varepsilon }_{0}$ are the dielectric constant of the medium and the vacuum permittivity, $R$ the AFM probe radius, ${\sigma }_{S}$ and ${\sigma }_{T}$ the surface densities of the samples and AFM probe, $d$ the probe-surface distance, and ${\lambda }_{D}$ the Debye length, defined as ${\lambda }_{D}={\left(\varepsilon {\varepsilon }_{0}{\kappa }_{B}T/{e}^{2}{C}_{0}\right)}^{2}$, where ${\kappa }_{B}$ is the Boltzmann constant, $T$ the absolute temperature, $e$ the elementary charge, and ${C}_{0}$ the electrolyte concentration [58]. Since the surface charge density of the e-PDA and the AFM probe are unknown, the values of the surface charge density of the AFM probe have been taken from the literature [59], performing the study under the same experimental conditions, i.e., using a silicon nitride AFM probe and adjusting the pH with HCl and KOH solutions.

**Fig. 3:**
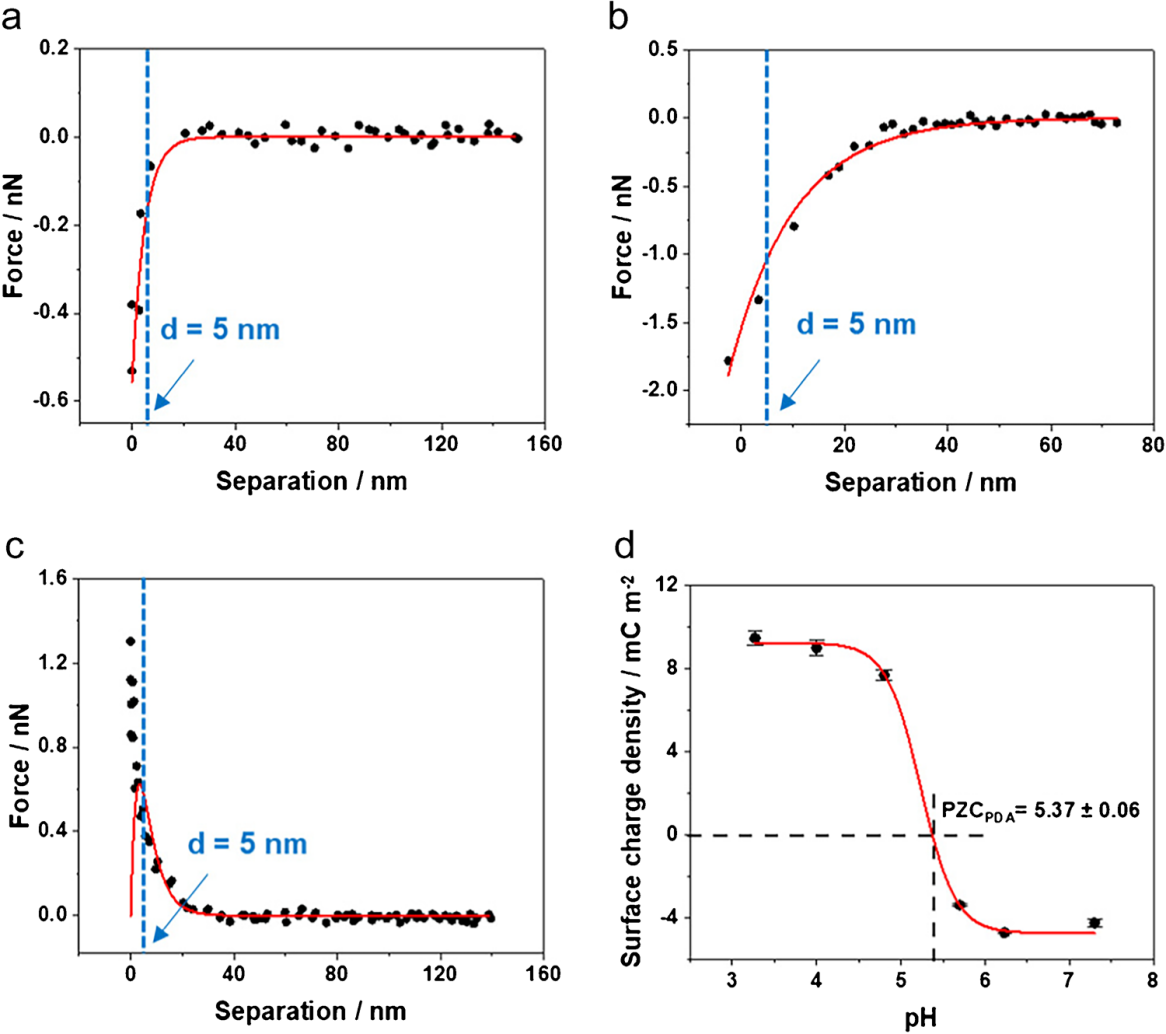
Fitting of exemplary force-distance approach curves assuming constant charge conditions at (**a**) pH 3.27, (**b**) pH 4.80, and (**c**) pH 7.30. (**d**) Effect of the pH on the surface charge density of e-PDA at 25 °C. Fitting parameters: curvature radius of the AFM probe $R$ = 20 nm, surface charge of the AFM probe − 0.010 C m^−2^ < ${\sigma }_{T}$<  + 0.015 C m^−2^ [59], Debye length 10^−7^ m < ${\lambda }_{D}$  < 10^−9^ m depending on the pH. For each pH value, 20 approach curves were used to extrapolate the surface charge density. The point of zero charge of e-PDA is also indicated

The approach curves (see Fig. 3) were obtained on e-PDA via AFM-based force spectroscopy and were fitted to the PBE assuming constant charge. This allows determining the surface charge density of e-PDA as a function of the pH. Representative force-distance approach curves (dotted lines) acquired on e-PDA and the corresponding fittings (red curves) are shown in Fig. 3a–c. To minimize the influence of short-range forces such as hydration forces (< 2 nm), which are not included in the mathematical model, the limit for the fitting was set to a tip-surface distance equal to 5 nm (blue dotted lines). The resulting pH dependency of the surface charge density of PDA is depicted in Fig. 3d. The obtained point of zero charge (PZC = 5.37 ± 0.06) is in good agreement with the ionic state of the functional groups of the e-PDA, and was confirmed by force titration. At a pH range between 3.5 and 5.7, indole and indoline groups of e-PDA are positively charged due to the presence of protons in the medium and are responsible for the charge regulation of the polymer. Thus, PDA is positively charged, as confirmed in Fig. 3d. At pH higher than 4.0, a slight decrease in the surface charge is evident. This behavior arises from the contribution of a minor amount of negatively charged carboxylate groups ($p{K}_{a}=$ 3.6), which may be generated during the electro-polymerization of dopamine [19, 41].

### Anti-biofouling studies on e-PDA

#### AFM force spectroscopy

The anti-biofouling properties of PDA are reported in literature mainly related to the presence of antimicrobial active functional groups, such as amine, amide, and radicals motifs [4, 5, 7, 60]. After the determination of the point of zero charge of e-PDA, these films were inoculated with *E. coli* suspensions (at a concentration of 10^8^ CFU mL^−1^) at three different pH values to investigate the adhesion behavior of bacteria grown at positively (pH 5), neutral (pH 5.5), and negatively charged (pH 7.0) e-PDA films. AFM imaging, AFM-based force spectroscopy, and ATR-FTIR measurements have been performed to characterize bacterial growth. pH values below 5 and above 7 have been excluded in this study to avoid bacterial stress due to extreme pH conditions.

The density of bacteria in contact with the e-PDA at different pH values does not show any statistically significant difference, as shown in the AFM images in Fig. S3.

**Fig. 4:**
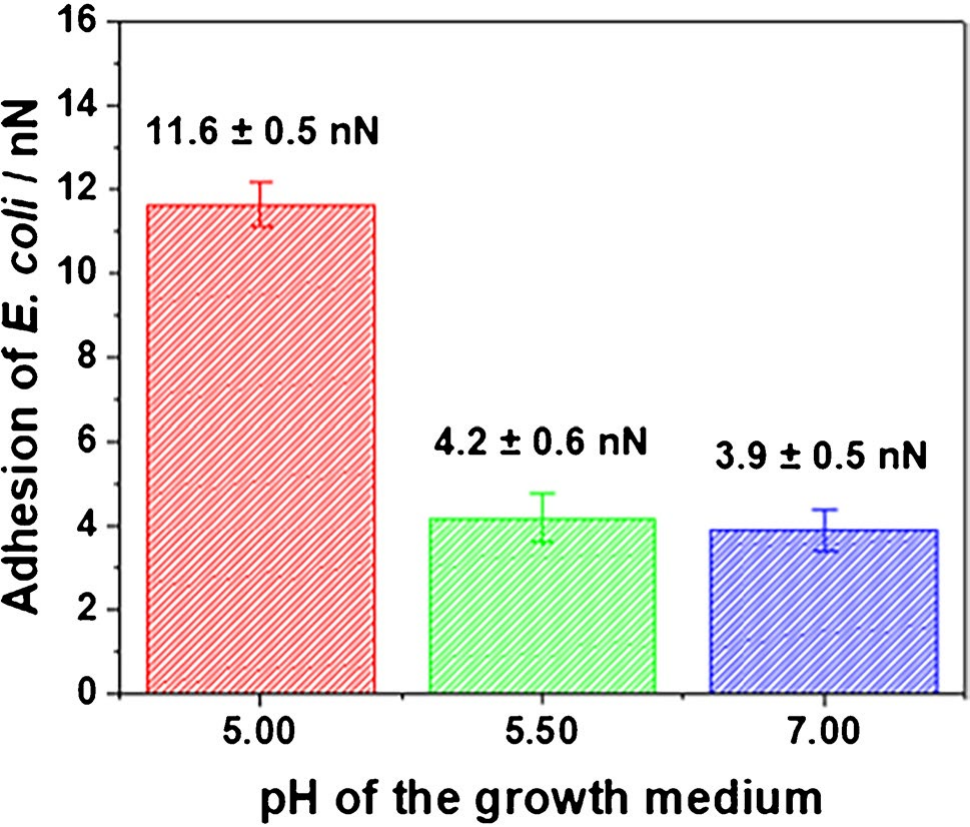
Bar chart of the measured adhesion forces of *E. coli* grown on e-PDA at different pH values (error bars reflect the measurements of at least 4 cells and 300 force curves)

Nonetheless, bacterial adhesion is strongly affected by the surface charge density of e-PDA. *E. coli* exhibits adhesion values of 11.6 ± 0.5 nN, if e-PDA is positively charged as shown in Fig. 4 (red bar chart). The cell adhesion values drop to 4.2 ± 0.6 nN, if e-PDA reaches the point of zero charge (Fig. 4 green bar chart). The rather high bacterial adhesion obtained at positively charged e-PDA is consistent with the intrinsic characteristics of the bacterial surface, which is known to be negatively charged. The negative charge of *E. coli* arises from the composition of the bacterial outer membrane (OM). The OM is a protective and selective barrier, characteristic of gram-negative bacteria, which surrounds the cytoplasmatic membrane [61]. One of the characteristics of the OM is the presence of phospholipids at the inner leaflet, and of lipopolysaccharides at the outer leaflet, which results in a net negative charge of the bacterial surface [61, 62]. Thus, the electrostatic attraction between the negatively charged cells and the positively charged e-PDA at pH close to 5 is responsible for the observed high bacterial adhesion. The adhesion is additionally increased by the hydrophobic nature of the bacterial OM and the e-PDA film [61]. *E. coli* grown on negatively charged e-PDA and neutral e-PDA exhibit similar adhesion forces as shown in Fig. 4 (green and blue bars). A decrease in adhesion force at negatively charged e-PDA was expected; however, a higher contribution of the attractive hydrophobic forces in comparison to the repulsive electrostatic forces between the bacteria and the e-PDA surface might take place, increasing the adhesion force. Thus, the electrostatic repulsion between *E. coli* and negatively charged e-PDA prevents bacterial attachment, while the hydrophobic interactions overcome the repulsive forces in turn promoting bacterial adhesion. Representative force curves recorded at *E. coli* are shown in Fig. S4.

**Fig. 5:**
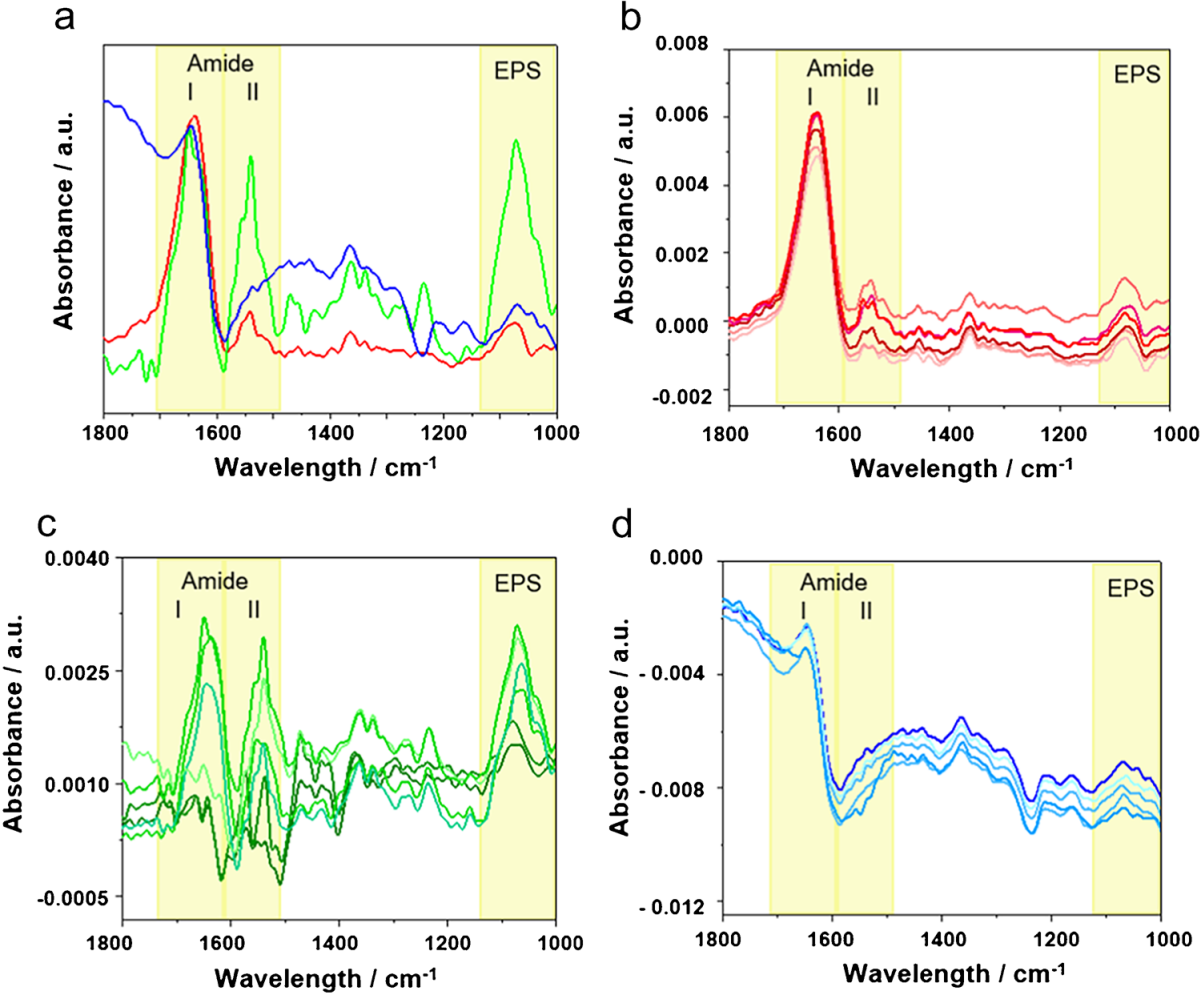
**a** ATR-FTIR spectra of *E. coli* biofilms grown at positively (red), neutral (green), and negatively (blue) charged e-PDA films. (**b**–**d**) ATR-FTIR spectra recorded at six different locations across an individual sample of *E. coli* grown at (**b**) positively (red), (**c**) neutral (green), and (**d**) negatively (blue) charged e-PDA surfaces. A 7-point FFT filter has been applied to all spectra; no baseline correction has been performed

#### ATR-FTIR studies

To validate the behavior of bacteria inoculated at differently charged e-PDA surfaces, ATR-FTIR experiments were performed. To evaluate only the vibrational signatures attributed to *E. coli* without overlapping features of e-PDA and LB medium, three additional e-PDA samples were prepared serving for recording background spectra that were only immersed in LB medium without bacteria present. Otherwise, these samples were treated the same way as the bacterial samples. It should be noted that using the e-PDA immersed in LB as background avoids the band overlapping not only of the LB medium, but also of PDA, which has characteristic vibrational features at ~ 1590 cm^−1^ (C = C of the aromatic ring) and at ~ 1650 cm^−1^ (C = N stretching imine and N–H bending amine vibrations) [15, 63], which may interfere with the amide I and amide II bands originating from the bacteria.

A qualitative comparison of the bacterial behavior at each e-PDA surface in dependence on the pH value is exemplarily shown by the three IR spectra in Fig. 5a. Furthermore, to ensure that the measurements were representative for the entire sample surface, for each of the e-PDA samples inoculated with bacteria, six different locations with a spot size of approx. 3 mm^2^ were investigated, as shown in Fig. 5b–d for the different pH values. To validate the presence of bacteria, three different spectral regions were evaluated: (i) the range between 1750 and 1600 cm^−1^ (amide I band), (ii) the range between 1600 and 1500 cm^−1^ (amide II band), and (iii) the range between 1140 and 1000 cm^−1^ (EPS region reflecting the presence of polysaccharides secreted during the biofilm formation [64]). The spectral range 1470–1380 cm^−1^ and 1270–1200 cm^−1^ (C = O symmetric stretching vibration indicating fatty acids and the presence of phospholipids) was not taken into account for the spectral interpretation herein, as these bands are not a direct indicator of biofilm formation and bacterial attachment [26].

The distinct appearance of amide I (1750–1600 cm^−1^) and amide II (1600–1500 cm^−1^) bands [65–67] suggests the presence of bacteria at the e-PDA surface. In this wavelength regime, C = O stretching vibrations (amide I) and out-of-phase N–H and C-N stretching vibrations (amide II) are located, and are characteristic of α-helix and β-sheet structures of proteins present within the bacterial cell membrane [65–67]. By comparing the amide bands at different pH values, it was shown that at the positively charged e-PDA film (Fig. 5b, red spectra), the amide bands are equally distributed across the investigated surface locations. At the neutral sample (Fig. 5c, green spectra), bacteria are apparently more inhomogeneously distributed, suggested by a change in intensity of the characteristic vibrational signatures generated by *E. coli* at the different investigated locations. At the negatively charged PDA sample (Fig. 5d, blue spectra), again, the bacteria seem to attach more homogeneously across the investigated areas of the e-PDA film. Furthermore, the spectra recorded at positively charged and neutral e-PDA samples reveal the presence of both amine I and II bands, while for the negatively charged e-PDA sample (Fig. 5a, blue line), the amide II band is absent or not detectable, yet amine I and EPS bands are present. Such a change in amide bands without correlated changes of the EPS band has been previously reported [26, 68–70], and has been suggested as a consequence of a loss of bacterial proteins due to a lack of nutrients or a hostile environment. In these situations, bacteria might consume inherent energy resources, i.e., their proteins to survive or start detaching from the surface. Consequently, the corresponding amide bands may decrease or disappear.

The spectral region 1140–1000 cm^−1^ (EPS band) encompasses the C = O, C = O-C, and P = O-C vibrations arising from carbohydrates, polysaccharides, and nucleic acids, which are associated with the presence of EPS secreted by the bacteria during adhesion and biofilm formation [65–67]. The EPS band is present in the spectra recorded at three inoculated e-PDA samples, and suggests that bacteria tend to form biofilms independently of the surface charge, albeit at different magnitudes. The evolution of the biofilm at negatively charged e-PDA appears favored by hydrophobic interactions between the bacterial cells and the surface leading to the formation of EPS. However, electrostatic repulsion due to negatively charged e-PDA may promote an early detachment of bacteria, and an associated decrease of the amide II band [71].

## Conclusions

In the present study, the adhesion properties of *E. coli* have been investigated in dependence of the pH using AFM-based force spectroscopy and force titration. For the first time, it was shown that the relation of the adhesion properties and the pH follows a double Boltzmann sigmoidal curve, from which an acidic dissociation constant of e-PDA equal to $p{K}_{a}$ = 6.3 ± 0.2 was derived. This value is in accordance with the proposed structure of e-PDA, which suggests the presence of functional groups such as indoline-quinones and indole-quinones, whose $p{K}_{a}$ is approx. 5.5. The investigation of the surface charge density of e-PDA in dependence on the pH led to a point of zero charge equal to 5.37 ± 0.06. Since the point of zero charge of a surface plays a significant role during bacterial attachment, the anti-biofouling properties of e-PDA were studied as a function of the pH, i.e., as a function of the surface charge density of the polymer. Adhesion force studies of *E. coli* grown at positive, neutral, and negative e-PDA films confirmed that the negatively charged bacterial cell wall of *E. coli* grown at positively charged e-PDA leads to higher adhesion forces in comparison to bacteria grown at neutral and negatively charged e-PDA. Finally, ATR-FTIR studies confirmed this trend indicating the spectroscopically more pronounced presence of bacteria at the positively charged polymer surface.

## Supplementary Information

Below is the link to the electronic supplementary material.

Supplementary file1 (PDF 1157 KB)
