# Supplementary material for: Atomic force and infrared spectroscopic studies on the role of surface charge for the anti-biofouling properties of polydopamine films
Source: Anal Bioanal Chem. 2022 Nov 24;415(11):2059–70. doi: 10.1007/s00216-022-04431-7 (PMC10079710; doi:10.1007/s00216-022-04431-7)
Supplement: Supplementary file 1 — Supplementary file1 (PDF 1157 KB) [file 216_2022_4431_MOESM1_ESM.pdf]

SUPPORTING MATERIAL

Atomic force and infrared spectroscopic studies on the role of surface charge for the anti-biofouling properties of polydopamine films

Giada Caniglia [a#], Andrea Teuber [a#], Holger Barth [c], Boris Mizaikoff [a,b], Christine Kranz [a*]

[a] Institute of Analytical and Bioanalytical Chemistry, Ulm University, Albert Einstein Allee, 11, 89081 Ulm, Germany
[b] Hahn-Schickard, Sedanstraße 14, 89077 Ulm, Germany
[c] Institute of Pharmacology and Toxicology, University of Ulm Medical Center, Albert Einstein Allee, 11, 89081 Ulm, Germany

[#] These Authors contributed equally.

* Corresponding author:
E-mail address: christine.kranz@uni-ulm.de

Content included in the Supporting Material:

S1. Current-time curve during the pulse deposition of e-PDA and cyclic voltammograms before and after the deposition

S2. Proposed pathways for the electrochemical polymerization of dopamine and final polymeric products

S3. AFM topography and deflection images of *E. coli* grown on negatively, neutral and positively charged e-PDA films

S4. Force curves recorded on *E. coli* grown on negatively, neutral and positively charged e-PDA films

S1. Current-time curve during the pulse deposition of e-PDA and cyclic voltammograms before and after the deposition

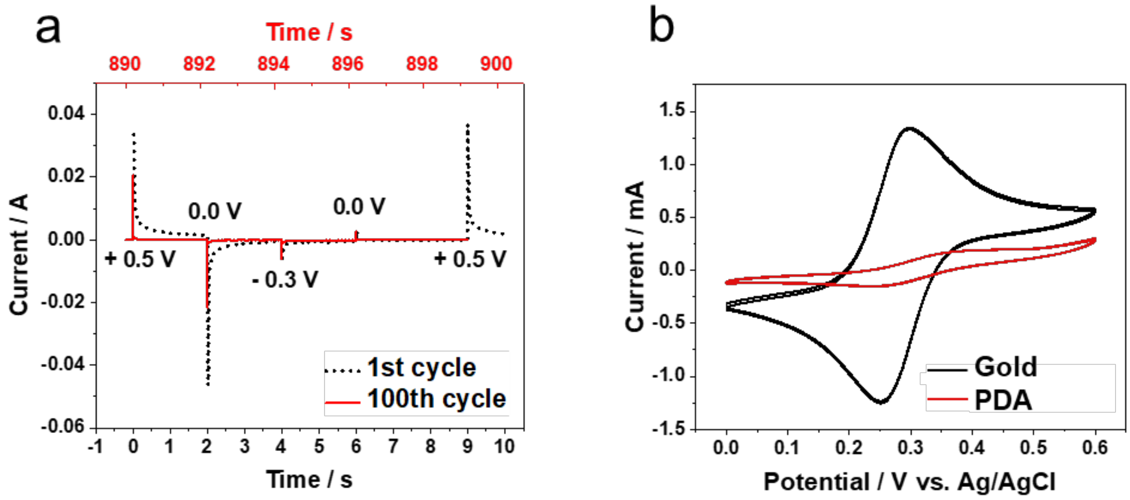

Fig S1 (**a**) Current-time curve during the pulse deposition of PDA: first cycle (black) and hundredth cycle (red). (**b**) CV recorded in 5 mM ferrocene-methanol and 0.1 M KCl before (Au substrate, black) and after PDA deposition (100-pulsed cycles, red); scan rate 0.05 V s^{-1}

S2. Proposed pathways for the electrochemical polymerization of dopamine and final polymeric products

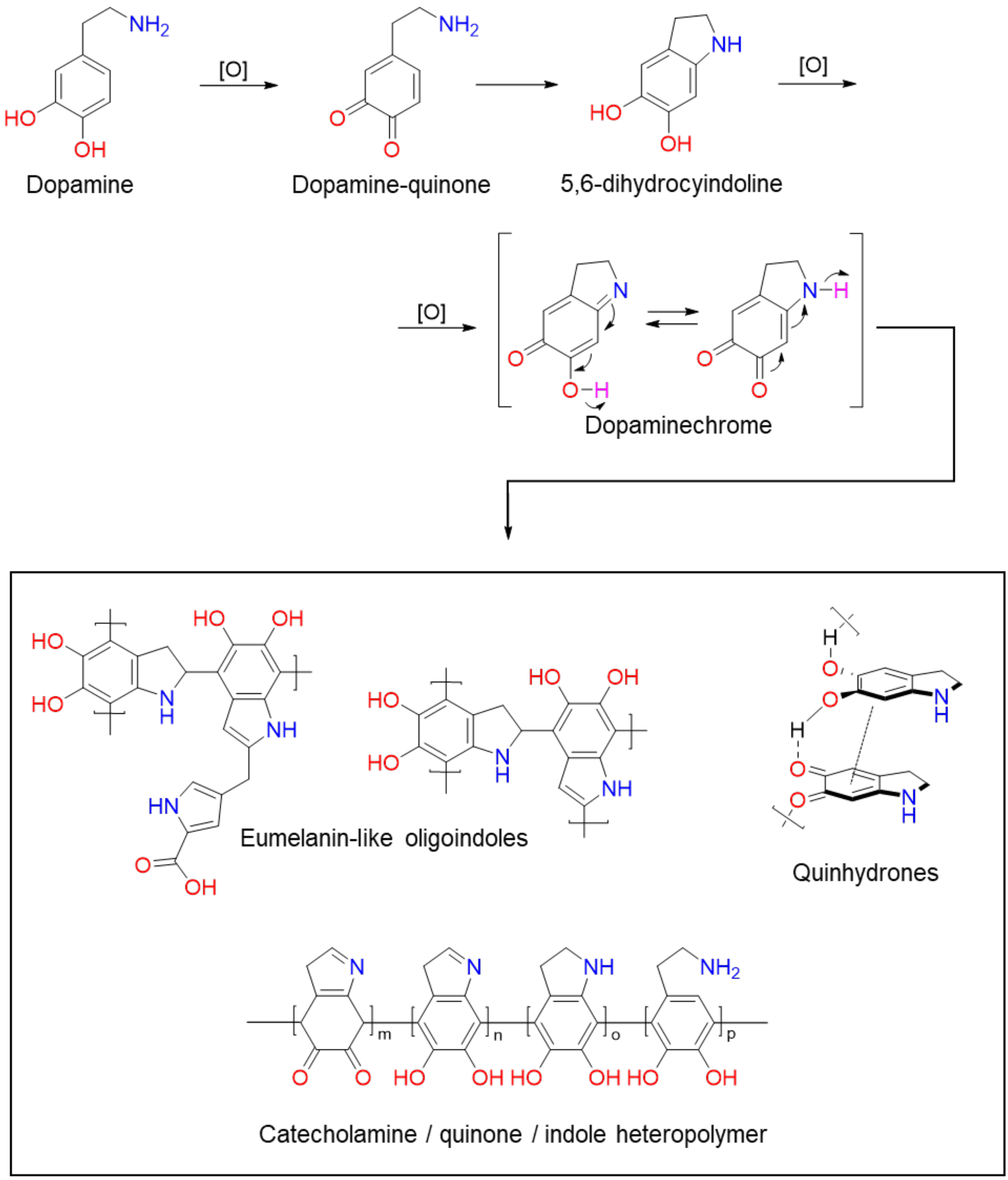

Fig S2 Proposed pathways for the electrochemical polymerization of dopamine and final polymeric products. Adapted from ref [1]

S3. AFM topography and deflection images of *E. coli* grown on negatively, neutral and positively charged e-PDA films

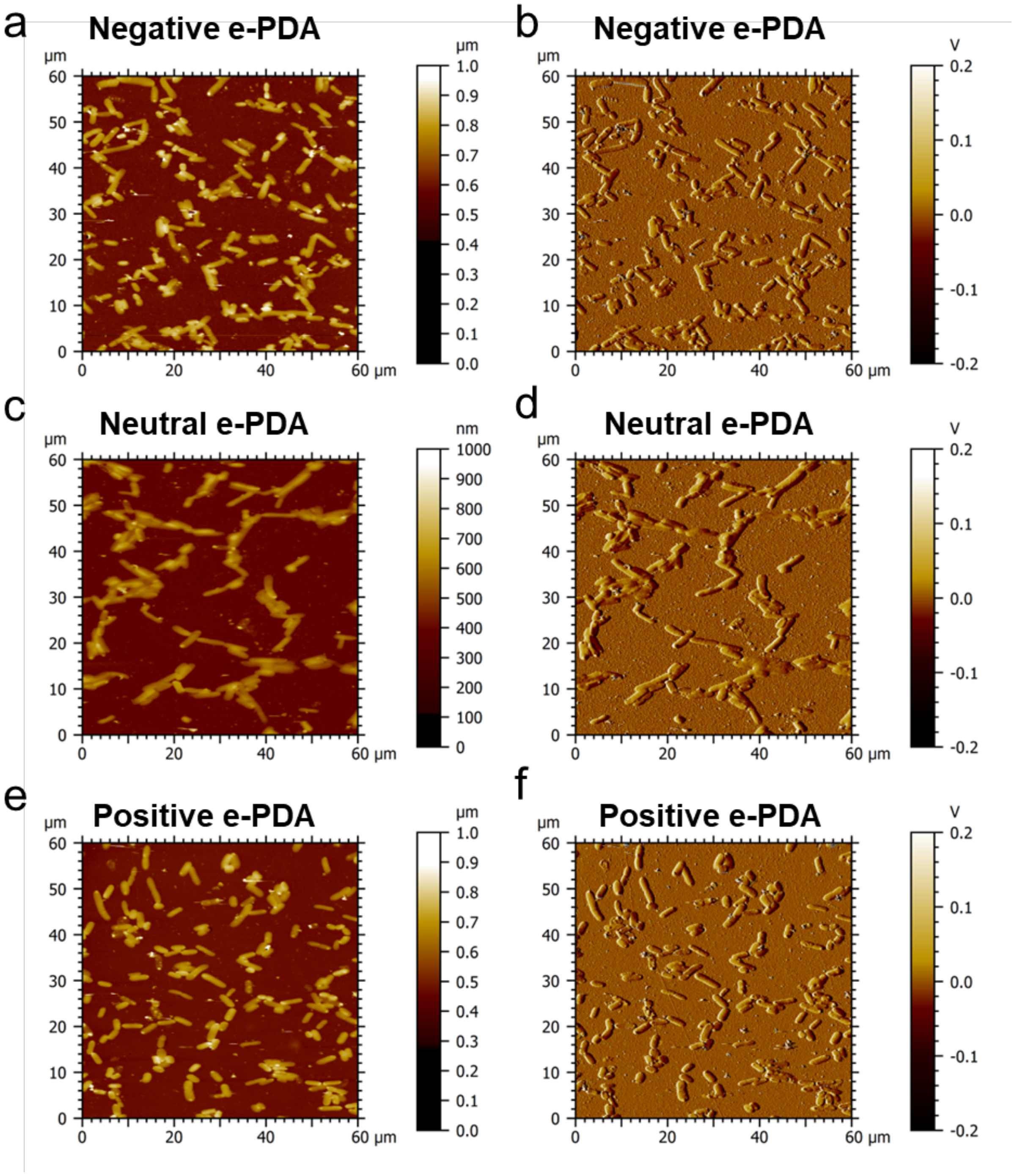

Fig S3. (**a,c,e**) AFM topography and (**b,d,f**) deflection images recorded in air of *E. coli* grown on (**a-b**) negatively charged PDA, (**c-d**) neutral PDA and (**e-f**) positively charged PDA. AFM contact mode images were recorded in air with silicon nitride probes (MLCT, Bruker AFM probes, CA, USA; nominal spring constant of 0.1 N m^{-1}) and a scan speed of 0.64 In s^{-1}

S4. Force curves recorded on *E. coli* grown on negative, neutral and positive e-PDA

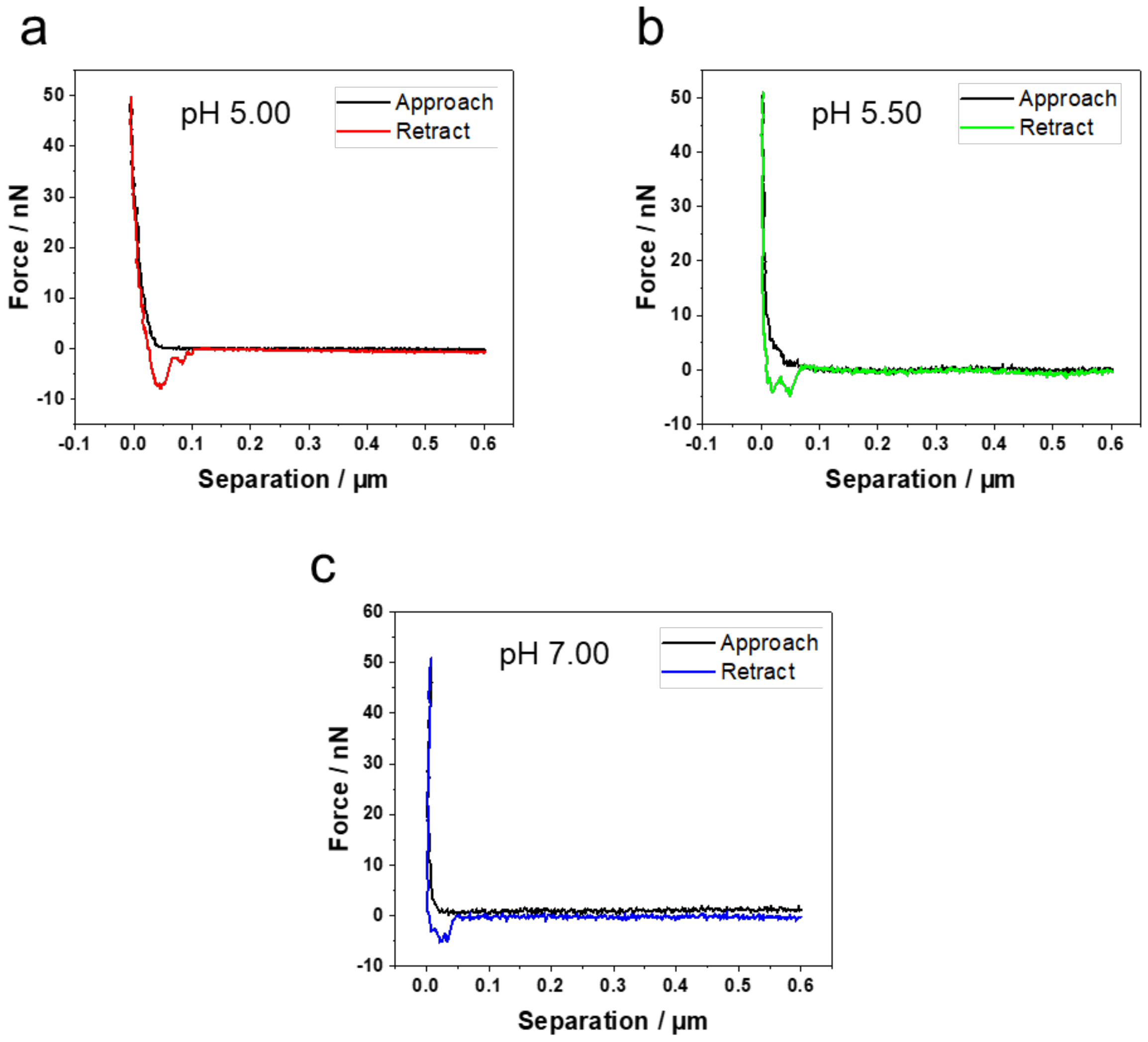

Fig S4 Exemplary force curves recorded on *E. coli* grown at (**a**) pH 5.00 (on positively charged PDA), (**b**) pH 5.50 (PDA close to the PZC) and (**c**) pH 7.00 (negatively charged PDA). Force-distance curves were recorded in solution using silicon nitride probes (MLCT, Bruker AFM probes, CA, USA; nominal spring constant of 0.1 N m^{-1}) with a sweep rate of 1.0 µm s^{-1} to minimize hydrodynamic effects and a loading force of 200 nN

References

[1] Lyu, Q.; Hsueh, N.; Chai, C. L. L. L. Unravelling the Polydopamine Mystery: Is the End in Sight? *Polym. Chem.* **2019**, *10* (42), 5771–5777. https://doi.org/10.1039/C9PY01372E.
